# Extreme weather effects on health services and communities in low and lower-middle income countries: a thematic systematic review

**DOI:** 10.1093/trstmh/trag007

**Published:** 2026-02-09

**Authors:** Julii Brainard, Yovitha Sedekia, Natalia R Jones, Michael Matte, Patrick Sunday, Deborah Watson-Jones, Daniel Dennis Mapemba, Severin A Kabakama, Edgar Mugema Mulogo, Moses Ntaro, Tran Thi Tuyet-Hanh, Paul R Hunter, Jo-Anne Geere

**Affiliations:** Norwich Medical School, University of East Anglia, Norwich NR4 7TJ, UK; Mwanza Intervention Trials Unit, National Institute for Medical Research, Mwanza, Tanzania; School of Environmental Sciences, University of East Anglia, Norwich NR4 7TJ, UK; Department of Community Health, Mbarara University of Science and Technology, P. O. Box 1410, Mbarara, Uganda; Department of Community Health, Mbarara University of Science and Technology, P. O. Box 1410, Mbarara, Uganda; Mwanza Intervention Trials Unit, National Institute for Medical Research, Mwanza, Tanzania; London School of Hygiene and Tropical Medicine, Department of Clinical Research, Faculty of Infectious and Tropical Diseases, Keppel St, London WC1E 7HT, UK; Kamuzu College of Health Science, Blantyre 3, Malawi; Mwanza Intervention Trials Unit, National Institute for Medical Research, Mwanza, Tanzania; Department of Community Health, Mbarara University of Science and Technology, P. O. Box 1410, Mbarara, Uganda; Department of Community Health, Mbarara University of Science and Technology, P. O. Box 1410, Mbarara, Uganda; Hanoi University of Public Health, No. 1A Duc Thang Road, Duc Thang Ward, North Tu Liem District, Hanoi 100000, Vietnam; Norwich Medical School, University of East Anglia, Norwich NR4 7TJ, UK; School of Health Sciences, University of East Anglia, Norwich NR4 7TJ, UK

**Keywords:** framework analysis, leadership, LMICs, storms

## Abstract

Most previous research about the dangers of extreme weather events was applicable to populations in high-income countries. Data summarising harms related to extreme weather events in low-income settings are lacking. A systematic review thematically summarising evidence about weather event-linked harms and responses in low- and lower-middle-income countries was conducted. Peer-reviewed and grey literature was systematically searched and selected. Data were extracted about harms, responses and outcomes relevant to six WHO building blocks of healthcare systems. Framework analysis was used to identify predominant themes related to harms, responses and the WHO building blocks. In total, 183 reports were included. Flooding and high winds were the most common types of extreme weather events documented. The main community experience themes identified were the displacement of populations and disruption. The main themes identified for health service delivery were vulnerability, disruption and resilience. Documented examples of resilience or recovery were far fewer for all six WHO healthcare system building blocks than descriptions of vulnerability and disruption. Extreme weather events can be highly disruptive and harmful to healthcare systems and communities in LMIC settings that are often already highly vulnerable.

## Introduction

Extreme weather events (EWEs) affect human health in many ways. Direct consequences may include injury, drowning or heat exhaustion. EWEs have the potential to damage infrastructure or pose risks to life. EWEs can lead to the destruction of property, transport and electricity networks, resulting in direct and indirect consequences on human experiences. In addition, the long-term indirect consequences from EWEs to human health include lower agricultural output affecting food security, ongoing disruption to healthcare services and access- and weather-related disruptions that undermine economic growth. The frequency of EWEs is thought to be increasing due to climate change.^1–3^

Health services in low- and lower-middle-income country (L&LMIC) settings are highly vulnerable to external shocks such as EWEs.^4–7^ Healthcare services in these settings frequently face challenges in the provision of high-quality healthcare, accessibility of services,^8–10^ negligible spare capacity in terms of staff, equipment, drugs and alternative facilities, patient populations with limited access to primary healthcare,^11^ inadequate budgets to maintain or repair buildings and equipment^9^ and chronic weaknesses in road,^12^ water^13^ and electricity networks.^14^ The addition of weather-related crises can exacerbate health service weaknesses. These challenges may combine to exacerbate negative outcomes following an EWE.

Syntheses describing the effects of EWEs on health services or human communities have mainly incorporated evidence from upper-middle or high-income country settings.^15,16^ Few studies have attempted to synthesise evidence about experiences only in L&LMICs and yet these areas are at high risk from EWEs. Here, we set out to thematically collate data about the effects of EWEs on healthcare services and wider human experiences in L&LMIC settings. This review elucidates the impact of these events on communities in L&LMICs, supplying knowledge that can be used to formulate targeted interventions. Further, this systematic review synthesises previous research to pinpoint areas with limited data or inadequate investigation, which could be used to direct research initiatives in L&LMICs.

## Methods

The protocols registered in association with this review at Prospero are CRD42024551416 and CRD42024551441. We designated any weather event described as negatively affecting health services or human communities as an EWE for that community, time and place. We use the word ‘reports’ to refer to all types of documents included in this systematic review, and from which we extracted data. The scope of a global review was potentially so huge that we would have assembled an evidence base far too large to ever process for synthesis. To make the review feasible, we therefore looked for any L&LMIC in the scientific literature search, while focusing parts of the grey literature search on just four exemplar lower-middle-income or low-income countries: Tanzania, Vietnam, Malawi and Uganda. Ethical approval was not required for this study because all the original data were already in the public domain and were intended to be highly accessible and available for synthesis.

### Inclusion criteria

The reports included had to describe impacts on health services or human experiences associated with a weather event. Natural disasters unrelated to weather, such as an earthquake or a tsunami, were ineligible. The EWE must have happened no earlier than 1990. Reports could be published in English, French, Vietnamese, Spanish or in a language that we could translate using Google Translate. The affected population or health service had to be located in a L&LMIC, according to World Bank 2022–2023 classifications at blogs.worldbank.org/en/opendata/new-world-bank-country-classifications-income-level-2022–2023.

Information found about any eligible country using the search strategy was eligible. Primary reports could be in any of these designs: grey literature, case study, cohort, cross-sectional, experimental, qualitative, systematic review. To be included, a systematic review had to have a research focus highly relevant to our own focus.

### Search strategy

The bibliographic databases MEDLINE and Embase (via OVID) and Scopus were searched on 27 August 2024, using the terms listed in Box 1.

Box 1. **Search terms used to find scientific literature.**

**(field=title) Weather words** = climate-change, cold, cyclon*, drought, *fire*, flood*, heat*, hurricane, land-slide, mudslip, mass-movement, rain*, *storm, summer, temperature, typhoon, weather, winter

**(field=title) Health care words** = care-service, clinic*, emergency-servic*, emergency-care, health-care, health-servic*, health-facilit*, health-cent*, health station*, health-sector, hospital*, medic*, nurs*, provider*

**(fields=title, abstract or keywords) Action words** = prepar*, recove*, read*, respons*, strateg*

**(fields=title, abstract or keywords) Impact words** = acute, chronic, close, effect*, impact*, isolation, outcome*, service-disruption, shutdown

**(fields=title, abstract or keywords) health system building-block key words** = delivery, workers, staff, nurses, carers, information-systems, digital-records, medical-records, medications, financ*, leadership, governance

The grey literature search was designed to minimise duplication. Grey literature reports often cite each other and thus can be highly repetitive. The grey literature search utilised Google searches, searches of the ReliefWeb archive and exemplar nation national document archives. The Google open search was for the first 40 results only, with no country names, using combinations of the keywords listed in Box 1. ReliefWeb was searched on 6 August 2024; other grey literature sources were searched during July–September 2024. Searches on ReliefWeb always included the exemplar country names. An important advantage of using a single main grey literature database (ReliefWeb) was to collect consolidated rather than duplicated information about a single event. ReliefWeb summaries are also narrative and thus appropriate for extracting data to be used in thematic synthesis.

### Screening abstracts and full text

De-duplicated bibliographic references were screened independently by two reviewers on the Covidence platform. Full-text versions of all the articles included by at least one reviewer were screened independently by two reviewers (Veritas Innovation Ltd, Melbourne, Australia). A third reviewer provided an opinion in cases of disagreements occurring during both screening stages.

### Data coding and extraction

Thematic data were extracted from articles not yet excluded after the full-screening stage. Extracted data were verbatim statements when brief (<30 words). Longer original statements were paraphrased into <30-word summaries. Where reports were structured with the relevant sections, only statements made in the Abstract, Discussion or Conclusion sections about the study’s findings were extracted. We focused on these sections to collect thematic rather than quantitative data. A single reviewer undertook initial extractions that were confirmed, revised and/or elaborated upon by a second reviewer.

In addition, we recorded bibliographic data, country location(s) of relevant populations or services and whether the statements described experiences or services. Aspects of what happened to health services were also coded for the six relevant WHO building blocks^17^: Health service delivery; Health services workforce; Health services information systems; Access to essential medicines; Health systems financing; and Leadership and governance.

### Thematic synthesis

Our aim was to synthesise data specific to EWE challenges affecting healthcare services and L&LMIC communities. We undertook framework analysis^18^ with respect to the extracted narrative data. Framework analysis is useful for answering a priori research questions or topics that relate to predefined areas of public health or health and social care services policy and practice. It is also useful for managing large datasets and is appropriate when a multidisciplinary research team are involved in analysis, and when data are drawn from diverse sources.^19^ The framework topics for health services synthesis were the six WHO building blocks. The framework topics for human experiences were two: Harms/Challenges that happened; and Response actions to the weather-linked crisis. Among the response actions, we also identified and describe in the Results section examples of these that appeared to work well.

To implement the synthesis, a brief summary of each original article’s content relevant to each topic was created. This was achieved in a matrix format, using a spreadsheet, with one row for each included report or study and the brief summary for each topic (columns). Summarising was undertaken separately for health services and other human experiences. PS, JG, JB, YS and MM created the matrices, which are available in Supplementary File S1 at https://osf.io/c95xq/files, and use colour coding to show how the extracted data were mapped to the final themes. These final themes are described in the Results section with examples.

### Quality assessment

The purpose of quality assessment in this review was to indicate the credibility and generalisability of the evidence sources. The results of the quality-assessment exercise consist of the numbers of reports that were judged to be either at least adequate or of high quality. Further details about the quality-assessment exercise undertaken in this review are available in Supplementary File S2 at https://osf.io/c95xq/files.

## Results

**Figure 1.**
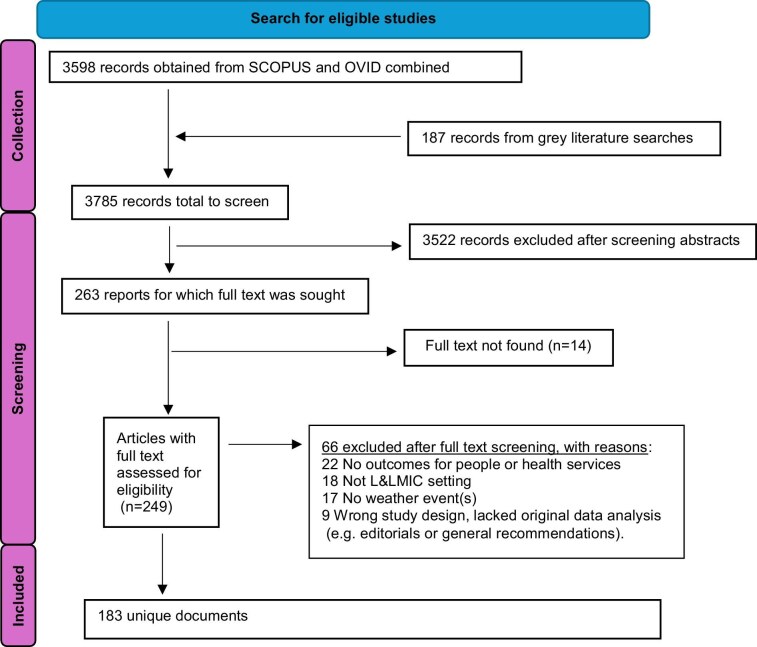
Study-selection procedure.

Figure 1 shows the report-selection procedure. Bibliographic details of the included reports are provided in Supplementary File S3 at https://osf.io/c95xq/files. In total, 183 reports were included. Table 1 lists the numbers of reports included by study design. Many reports provided data about both health services and human experiences, and most were grey literature. Table 1 also lists the numbers of reports that were considered adequate or of high quality. Most reports were judged to be of at least adequate quality, although only a minority were regarded to be of high quality.

**Table 1.** Number of reports providing data about health services and/or human experiences, by design

Report design (no.)	Adequate quality	High quality
Case study (19)	13 (68%)	3 (16%)
Cohort (11)	9 (82%)	2 (18%)
Cross-sectional (15)	15 (100%)	7 (47%)
Experimental (2)	0 (0%)	0 (0%)
Grey literature (124)	107 (86%)	24 (19%)
Qualitative (6)	6 (100%)	6 (100%)
Systematic review (6)	4 (67%)	1 (17%)

Note: All high-quality reports are subsets of the ‘adequate’ quality reports, for each study design. The descriptors are defined thus: ‘adequate quality’ reports had at least one reviewer who answered ‘Yes’ to at least 67% of quality-assessment questions. A report was designated ‘high quality’ when both reviewers answered ‘Yes’ to at least 67% of quality-assessment questions.

The most mentioned countries were Vietnam (n=38), Uganda (30), Philippines (35), Tanzania (13), Mozambique (12) and Malawi (10). Most reports (n=166) were about an event that was described as a typhoon, storm, cyclone or heavy rains. Flooding was the dominant mechanism causing harm. Fifteen reports described high temperatures, 13 drought, three lightning, while two were not specific about the type of EWE. The median year of publication was 2019.

In the Results summaries below, up to three data sources are provided to support specific statements; individual sources that contributed to each thematic summary are listed in Supplementary File S1 at https://osf.io/c95xq/files.

### Human experiences: harms that happened

#### Damage to homes and infrastructure

Most of the reports included (n=139/183) described damage caused by EWEs to infrastructure and/or property. This damage often resulted in people being evacuated from their homes.^20,21^ Damage to road, electricity and telephone networks was often documented.^21^ Many displaced persons sheltered in public buildings, especially schools, while others stayed with family or friends. Flooding sometimes affected latrines, destroying community facilities or leading to contaminated homes or crops.^22,23^ On occasion, safe water supplies such as boreholes were inundated with unsafe floodwater, leading to drinking water scarcity and sanitation risks.^24,25^

#### Threats to health and livelihoods

Injuries, exacerbations of chronic illness, deaths and harms to livelihoods were described in 159 reports. Flooding often led to crop losses, which then contributed to both income loss and the risk of food insecurity.^26^ Floods often damaged schools, leading to loss of education and the loss of potential shelter locations for displaced persons.^21^ Higher hospital admissions were associated with heatwaves,^27^ as was an increased risk of kidney injury.^28^ Both physical and mental trauma among displaced persons and EWE survivors was recorded.

### Human experiences: response actions

#### Community-based support

Community-based support was a theme in 57 reports. Immediate response actions were usually organised by those communities directly affected by EWEs, especially after flooding. Schools and churches often became evacuation centres.^29^ Rescue of friends and neighbours was sometimes necessary at short notice. Support for affected people included the provision of food, hygiene supplies, safe drinking water, land to set up camps, cash and other equipment or assistance. Warning systems—to the extent that they existed at all—were relatively local and tended to be informal.

##### What worked well.

The nature of community responses is that they are often ad hoc, difficult to observe and poorly documented. However, local or international organisation and response planning was sometimes documented as effective. For instance, Save the Children^30^ described how Village Disaster Management Committees led community responses during floods, and mobilised and coordinated relief aid and rapid assessments.

#### Government and non-governmental organisation support

Government and non-governmental organisation (NGO) support, which emerged as a theme in 61 reports, mainly occurred after community self-help began. Governments and NGOs were well placed to provide extreme weather warnings before an EWE.^31,32^ After EWEs occurred, governments and NGOs were able to coordinate deployments of recovery and emergency healthcare teams,^33–35^ provide food and other aid^36–38^ and produce professional needs assessment summaries,^22,39,40^ appealing for international or donor aid.

##### What worked well.

Local community capacity to provide emergency food, hygiene equipment and places to sleep tended to be limited. Larger external organisations had important roles in ensuring that the needs of displaced persons were met beyond the acute crisis phase. NGOs and governments were especially able to provide or facilitate support in the form of coordinating or providing emergency response and medical teams, expert input to restoring healthcare systems, sanitation and infrastructure repairs, agricultural recovery and rebuilding homes. The achievement of these outcomes was typically preceded by a formal needs assessment.

### Health service delivery

Under the WHO building block of Health service delivery, from 82 reports we identified three key themes: vulnerability, disruption and resilience.

The vulnerability of pre-existing services and/or the population being served by health services was described in 47 reports. Clinical buildings and sites were often damaged by flooding, landslides or high winds.^41–43^ Populations were vulnerable because of occupational exposures^28^ or chronic illness.^44^ Higher numbers of hospital admissions^45^ and kidney injuries^46^ were sometimes associated with heatwaves. Postdisaster trauma injuries were likely, especially among men, during postdisaster clean-up activities.^47^ Flooding might mean the contamination of land, homes and food crops, with faecal matter increasing the risk of disease. Displacement from homes was often traumatic and undermined the mental health of residents,^44^ while staff were at risk of psychological distress due to high occupational demands (see the ‘Health services workforce’ section below).^38,48,49^

Disruption was described in 42 reports and, most typically, indicated significant delays rather than a cessation of services. Meanwhile, healthcare buildings and equipment within these buildings might be destroyed, badly contaminated or damaged. In addition, road network damage meant that patients could not travel to access healthcare, floods might wash away those medications needed to manage chronic conditions and vaccination programmes were paused while other emergency care needs were addressed.

Regarding resilience, the baseline quality of services was rarely described as good. However, examples of especially poor or relatively good practice were described. For instance, Hansson et al.^28^ described a programme to educate sugar-cane cutters as to how to reduce fluid loss and the risk of kidney injury. Labarda et al.^50^ described the reasons for differences in resilience for hospitals affected by a severe typhoon. They identified eight domains in which a more resilient hospital outperformed local hospitals that were otherwise more overwhelmed by a typhoon: leadership, preparedness plans, stockpiling, safety, critical care, staff training and recovery plans. In general, more rapid mobilisation of community and NGO/government personnel was associated with more rapid recovery to pre-existing service levels.

### Health services workforce

For the WHO Health services workforce building block, we identified staff shortages, training issues and staff well-being as primary themes in 34 reports.

Staff shortages were common, especially during the acute phase of any weather-linked crisis, not least because staff could not travel on damaged roads, a situation which hindered travel for everyone,^51^ while the homes of staff might also be damaged and their own families put at risk.^52,53^

Training of staff what to do in cases of weather-linked service disruption, was either not done or was inadequate in many reports.^54,55^

Staff well-being could be undermined when staff were worried about their own families, friends and communities. Staff being highly committed to providing care, but also exhausted by their work, was documented.^49^

### Health services information systems

From 19 reports, we identified the primary themes for the WHO Health services information systems building block to be the benefits and challenges of using data.

Regarding benefits, several reports documented how complete records of patient presentations made it possible to optimise clinical staff deployment^56,57^ to where need was greatest. Medical records also supported timely care for staff themselves, by tracking the risk of mental distress or other illness among staff providing care in communities affected by or recovering from EWEs.^58^

Challenges in medical record-keeping and processing arose from poor-quality record-keeping at baseline, as well as damage to infrastructure, such as the loss of physical records and electricity or mobile phone networks going down or becoming especially unreliable.^47,52^

### Access to essential medicines

The themes identified for the WHO Access to essential medicines building block consisted of descriptions of disruption, as well as recovery steps, in 30 reports.

Disruption and shortages were often immediate. Medications within facilities were not always stored above inundation zones.^52^ Access to stores might be hindered by damage to buildings and an inability to deliver medications to patients, who themselves were unable to travel due to damaged roads. Inadequate financing led to minimal stockpiling,^59^ which meant poor reserves if demand should surge, for example if a large number of patients had their medications washed away at home by the flood. Flooding itself could result in both water and the environment becoming contaminated, resulting in an increased risk of contagious diseases and thus increased for medications that were not routinely stockpiled.^38^

Regarding recovery, there were some examples of a fast resupply of essential medicines,^35,57^ especially when coupled with a formal needs assessment, as well as programmes to re-establish medication adherence by patients.^60^ A disrupted vaccination programme was described with the necessary steps and timeline for returning to previous inoculation delivery rates.^35^

### Health services financing

Two main themes were identified for the WHO Health services financing building block: adequacy of funding and coordination with needs assessment (or not). Data came from 17 reports, which was the smallest evidence base for any of the WHO building blocks used to structure our synthesis.

Funding at baseline and during recovery periods was most often described as inadequate.^54,61^

Some reports addressed coordination or lack thereof regarding the allocation of funds or response teams with or without formal needs assessments. Some reports tried to assess if the money spent on health services recovering from EWEs had been an effective and good use of resources.^62^

### Leadership and governance

The themes identified for the WHO Leadership and governance building block were coordination and implementation, which respectively reflected the potential objectives of good leadership and good governance in 37 reports.

Regarding coordination, leaders are likely to have authority and the opportunity to organise others, and thus coordinate prevention, reduction or mitigation strategies, aid and support. The most valuable coordination actions described in the included reports were good quality engagement with relevant stakeholders, utilisation of credible power structures and honest and clear communications.^48,57,63^

The implementation theme encompassed aspects of how response actions occurred or should happen to support health services. Often the content consisted primarily of recommendations,^56,57^ although some successfully tested practices were mentioned. These included staff learning how to drive tractors to reach vulnerable patients^64^ and communication channels that facilitated the sharing of important resources between response teams.^47^

## Discussion

EWEs can be highly disruptive and harmful to healthcare systems and communities in L&LMIC settings.

This systematic review benefitted from a broad search and selection strategy and the perspectives of coauthors in high-, low- and lower-middle-income countries. A large amount of grey literature was included that documented harms, actions and needs as they occurred, including localised and specific needs assessments. The inclusion of grey literature was feasible because we focused the grey literature search on four countries: Malawi, Tanzania, Uganda and Vietnam.

We are not aware of any previous systematic reviews that document all types of community experience in L&LMICs in relation to all categories of EWEs. We found more evidence about health services than community outcomes. Storms were the dominant EWE in our review. Most previous research about EWEs and healthcare systems concerned populations in high- or upper-middle-income countries, and mostly documented the effects of heatwaves and not storms.^15^ The type of EWE has an important influence on the types of patient presentation. Dominant types of presentations or service disruptions associated with heatwaves are unlikely to be similar to the types of presentations and disruptions associated with flooding, displacement from homes or high winds linked to storms. The structure of health services and their inherent capacity to respond and recover is likely to be very different between high- and much lower-income country settings. Also, within L&LMICs especially, relatively local resources, including community health workers (CHWs), may be important. CHWs may have unique opportunities to provide an effective first response to EWEs in L&LMIC communities and to health services.^65^

With respect to community experiences, a summary interpretation of our results is that disruption to daily life due to EWEs has been high in many cases. People had to leave their homes, at least temporarily, food and water insecurity increased and infrastructure was often badly damaged.

With respect to health services, our review suggests a picture of continuing but very highly disrupted service delivery after EWEs. Staff were often highly committed but vulnerable, like their surrounding community, to risks of displacement and concerns about their own family’s safety. Medication shortages were common after EWEs. Good health information systems often helped health services to recover quicker and effectively target limited resources. Leadership that encouraged multiagency collaboration and formal needs assessments seemed to help aid and other resources to be delivered faster to where they were needed.

Opportunities will need to continue to be sought to build resilience in communities and health services in L&LMICs affected by EWEs. Gkouliaveras et al.^16^ recommended that future longitudinal studies considering the impacts of climate change on health systems should focus on identifying beneficial strategies that incorporate adaptation, mitigation, preparedness and recovery planning.

A key vulnerable group in L&LMIC settings, with regard to weather-linked hazards, are residents of ‘unplanned settlements’ within urban areas. Unplanned settlements typically become established on land not allocated for development because it is inherently prone to landslides or flooding. Because of their origins, these neighbourhoods also often lack infrastructure such as electricity, running water or sanitation. In L&LMICs, a high proportion of the population live in unplanned settlements,^66^ but there is a paucity of research about effective climate change-adaptation strategies in such neighbourhoods.^67^ Effectively responding to and minimising health impacts arising from EWEs that affect unplanned settlements may require an intersectional approach.^4^

Health services that are insufficiently resourced for ‘normal’ circumstances will be especially unprepared for EWEs. Our synthesis suggests there is a need for greater planning and resource provision across the WHO building blocks. Our data about community experiences before, during and after EWEs provide similar evidence that greater planning, coordination and resource provision is needed to reduce vulnerability and increase the resilience of both health services and the communities they serve in L&LMICs.

The limitations in our study include that we did not explore between-country or between-region differences. There were 77 L&LMICs listed by the World Bank in 2024 (source = https://datacatalogfiles.worldbank.org/ddh-published/0037712/DR0090755/CLASS.xlsx). These countries may vary significantly in their vulnerabilities, resilience and capacity for coping or adaptation. We have not attempted to capture these nuances. Our grey literature search was made feasible by making it finite, which means that the grey literature search had a primary source (i.e. ReliefWeb) and a partial focus on specific exemplar countries.

## Conclusions

Extreme weather events have widespread effects on communities and health services in L&LMIC settings. There are examples of resilient and effective responses to weather-linked harms, as well as documented instances of poor-quality responses and inefficient practices. The evidence provided in this review can be used to inform health services policy, planning and preparation for EWEs, which are predicted to become more frequent and severe with climate change.

## Supplementary Material

trag007_Supplemental_Files

## Data Availability

The data matrices are available as Supplementary File S1 at https://osf.io/c95xq/files.
