# Supplementary material for: Extreme weather effects on health services and communities in low and lower-middle income countries: a thematic systematic review
Source: Trans R Soc Trop Med Hyg. 2026 Feb 9;120(7):723–31. doi: 10.1093/trstmh/trag007 (PMC13339161; doi:10.1093/trstmh/trag007)
Supplement: trag007_Supplemental_Files [file trag007_supplemental_files.zip › S2 Quality Assessment Description.docx]

### Quality Assessment

The purpose of quality assessment in this review was to indicate credibility and generalisability of the evidence sources. The results of the quality assessment exercise were counts of studies that were judged to be relatively at least adequate or high quality.

We modified existing checklists appropriate to each study design to assess study quality. When the study design was cohort, cross-sectional, experimental, qualitative or systematic review, our quality assessment questions were based on CASP checklists (https://casp-uk.net/casp-tools-checklists/). The checklist modified for grey literature quality assessment was the AACODS checklist ^1^. The original quality checklist modified for case study designs was from the Center for Evidence Based Management ^2^. Most of the original checklist questions were designed to be answered as yes/no/can’t tell. Where it was possible to answer the original checklist question with a yes or no answer we used the original instrument question; some questions were changed to enable answers that could be yes/no/can’t tell/not applicable. The checklist questions that we used are shown in Supplementary file S3 which also shows the quality assessment answers given.

Two researchers undertook quality assessment independently. The original and modified questions were subjective with many criteria to consider. Questions were not comparable between study designs because, for instance, best practice when undertaking experimental study designs is different from best practices in conducting qualitative studies or producing grey literature. However, regardless of study design, each quality assessment question was designed such that the preferred answer would be Yes. Preferred answer in this context was meant to best practice, with respect to that study design, indicating maximum credibility, minimal bias, generalisable results, best scientific practice, most transparent conduct or sufficient relevance to L&LMICs and wider understanding of resilience of health care systems to weather events. We report the quality assessments after assigning a description of ‘high quality ‘ to a study if both reviewers gave ‘Yes’ answers to at least two thirds of questions about that study, and a description of ‘adequate quality’ if at least one reviewer gave ‘Yes’ answers to at least two thirds of questions about that study. We did not attempt reconciliation between the reviewer answers because of the high degree of subjectivity implicit in answering most of the instrument questions. Quality assessment results were also separated by study design.

References:

1. Tyndall J. *AACODS checklist*. <https://policycommons.net/artifacts/4855940/untitled/5692885/> (Date Accessed 2010 Accessed, date last accessed)

2. Center for Evidence Based Management. *Critical Appraisal Checklist for a Case Study*. <https://www.cebma.org> (Date Accessed 2014 Accessed, date last accessed)
